# Study on the adsorption properties of Cr(vi), Cu(ii) and Cd(ii) in acid mine wastewater by coal gangue loaded nano-FeS

**DOI:** 10.1039/d5ra04083c

**Published:** 2025-08-05

**Authors:** Xuying Guo, Wei Sun, Honglei Fu, Zilong Zhao, Xiaoyue Zhang, Fanbo Meng, Yanrong Dong

**Affiliations:** a College of Science, Liaoning Technical University Fuxin 123000 Liaoning China guoxuying@lntu.edu.cn +86-24-13941834560; b College of Environmental Science and Engineering, Liaoning Technical University Fuxin 123000 Liaoning China; c College of Civil Engineering, Liaoning Technical University Fuxin 123000 Liaoning China; d College of Mining, Liaoning Technical University Fuxin 123000 Liaoning China

## Abstract

This study prepared a novel composite material, coal gangue loaded nano-FeS (nFeS-CG), to remove Cr(vi), Cu(ii) and Cd(ii) from Acid Mine Wastewater (AMD). The system evaluated the synchronous removal effect of nFeS-CG on Cr(vi), Cu(ii), and Cd(ii) in AMD. The effects of nFeS-CG dosage, initial pH, reaction time and initial concentration on the adsorption of Cr(vi), Cu(ii) and Cd(ii) in AMD were investigated. The adsorption mechanism of nFeS-CG for Cr(vi), Cu(ii) and Cd(ii) in AMD was studied by adsorption isotherms, adsorption kinetics and adsorption thermodynamics models, combined with SEM, XRD, FTIR and XPS. The optimum conditions for the adsorption of Cr(vi), Cu(ii) and Cd(ii) in AMD by nFeS-CG were dosage of 7 g L^−1^ and reaction time of 155 min. The best removal rates were 94.08%, 84.08% and 71.36%, respectively. The adsorption of Cr(vi), Cu(ii), and Cd(ii) by nFeS-CG conformed to the Langmuir model and the pseudo-second-order kinetic model, indicating that the adsorption process was monolayer adsorption and was dominated by chemical adsorption. The thermodynamic results showed that the adsorption process was an endothermic process, and the temperature rise was beneficial to the adsorption. The characterization results and mechanism analysis showed that the removal mechanism of Cr(vi), Cu(ii) and Cd(ii) by nFeS-CG was mainly electrostatic adsorption, redox and precipitation. Co-existing ions, leaching toxicity and recycling experiments proved nFeS-CG's strong practical application potential as a low-cost adsorbent for simultaneous removal of multiple heavy metals in AMD. This study provides technical reference for coal gangue utilization and AMD remediation.

## Abstract 2

This study prepared a novel composite material, coal gangue loaded nano-FeS (nFeS-CG), to remove Cr(vi), Cu(ii) and Cd(ii) from Acid Mine Wastewater (AMD).

## Graphical abstract

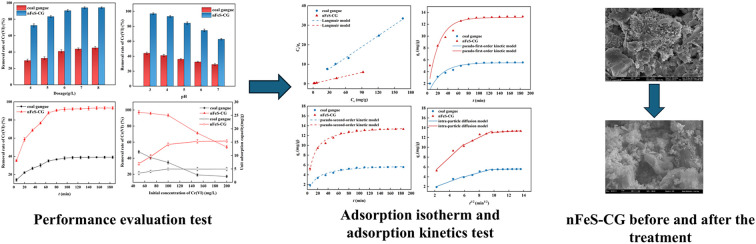

## 1. Introduction

With the rapid development of industry and the continuous development of mineral resources, a large number of mining activities have led to the destruction of mine ecological environment, and the pollution of acid mine wastewater is becoming more and more serious.^1–3^ With the increase of AMD emissions, its harm to the environment is becoming more and more significant. AMD contains high concentrations of copper, chromium, cadmium and other heavy metal ions,^4^ These heavy metal ions are difficult to degrade and bioaccumulate, posing a serious threat to the ecological environment and human health.^5^ At present, the common methods for treating heavy metal pollution in AMD include microbial method,^6^ membrane separation method,^7^ ion exchange method,^8^ adsorption method^9^ and chemical precipitation method.^10^ Among them, chemical precipitation method is widely used in the treatment of heavy metal ions in AMD due to its advantages of simple operation and high treatment efficiency. However, the stability of precipitates produced by common chemical precipitants (such as lime, caustic soda, soda ash and sodium bicarbonate) is poor, which limits their practical application.^11^ Therefore, it is necessary to find a material with high treatment efficiency and stable precipitation.

Nano-FeS is widely used in the treatment of heavy metal ions in AMD due to its strong acid resistance, high reducibility, and the ability to quickly reduce heavy metal ions and combine them to form stable sulfide precipitates. Chen^12^*et al.* used biosynthetic nano-FeS to treat Cu(ii) in AMD, and the data revealed that nano-FeS had an 87% Cu(ii) removal rate in AMD. Kim^13^*et al.* successfully prepared nano-FeS particles using disulfite. However, the prepared nano-FeS tends to agglomerate and oxidize easily due to its high surface energy, which affects its adsorption performance. Thus, it is critical to identify a carrier material that enhances the dispersion and stability of nano-FeS. Recent studies have shown that mineral materials as carriers can effectively inhibit the agglomeration of nano-FeS.^14^ Jia^15^*et al.* used kaolinite-loaded nano-FeS to treat Cr(vi) in simulated heavy metal wastewater. The experiment showed that the maximum adsorption capacity of Cr(vi) was 45 mg g^−1^, it was confirmed that kaolinite-loaded nano-FeS can significantly enhance the removal of heavy metal chromium (Cr). Lian^16^*et al.* used attapulgite-loaded nano-FeS to adsorb Mo(vi). The results showed that the removal rate of Mo(vi) was 83%, but its preparation cost was high and it was difficult to achieve large-scale application. Therefore, it is necessary to further screen cheap and stable carrier materials.

Coal gangue is an industrial solid waste generated during coal mining and processing,^17,18^ massive stacking not only affects the utilization of surrounding land, but also easily causes spontaneous combustion.^19^ Therefore, the resource utilization of coal gangue urgently needs to be solved. Li^20^*et al.* explored the removal of Cr(vi) from wastewater by coal gangue, experiments confirmed that coal gangue has a certain reduction ability to Cr(vi) in water. However, the adsorption capacity of coal gangue is generally not high when it is used alone, and it needs to be modified to improve the adsorption efficiency. Wang^21^*et al.* successfully prepared coal gangue loaded Fe/FeO_*x*_ nanoparticle composites by liquid phase reduction method, and the adsorption capacity of Cd(ii) in wastewater reached 149.53 mg g^−1^. This shows that coal gangue loaded nanoparticles can effectively remove heavy metal Cd(ii). The structure of coal gangue is stable, which can be used as a mineral skeleton to control the size of nanoparticles and increase the mechanical support force and thermal stability. Therefore, it can be considered to select cheap and stable coal gangue as the carrier material to load nano-FeS to improve the treatment effect of heavy metal wastewater.

Based on this, in this study, the nano-FeS adsorption material loaded on coal gangue was prepared by ultrasonic precipitation method, which not only enhanced the adsorption performance of coal gangue, but also improved the agglomeration of nano-FeS, and realized the synergistic optimization of material properties. It was applied to treat Cr(vi), Cu(ii) and Cd(ii) in AMD. The study examined how adsorbent dosage, initial pH, reaction time, and initial solution concentration influence the adsorption of Cr(vi), Cu(ii), and Cd(ii) by nFeS-CG. The mechanism of Cr(vi), Cu(ii) and Cd(ii) removal by nFeS-CG was revealed by adsorption kinetics, adsorption isotherms, adsorption thermodynamic model, XRD, SEM, FTIR and XPS. Through the application of coal gangue loaded nano FeS material, it provided a certain technical reference for the comprehensive utilization of coal gangue.

Compared with previous studies on FeS-loaded materials, this work innovatively utilizes coal gangue as a carrier, which not only reduces costs but also enhances the dispersion of nano-FeS through ultrasonic precipitation. Moreover, nFeS-CG achieves synchronous removal of Cr(vi), Cu(ii), and Cd(ii) *via* combined mechanisms (electrostatic adsorption, redox, and precipitation), addressing the limitation of single-metal treatment in most existing adsorbents.

## 2. Materials and methods

### 2.1 Experimental materials

#### 2.1.1 Coal gangue

The coal gangue used in this experiment was collected from an abandoned coal mine in Fuxin City, Liaoning Province, China. The jaw crusher and the roller crusher were used to crush the coal gangue, and the coal gangue samples with a particle size range of 0.106–0.125 mm were selected. The samples were washed three times and dried for later use. The major chemical constituents of coal gangue are listed in Table 1.

**Table 1:** Main compositions of coal gangue

Component	SiO_2_	Al_2_O_3_	TiO_2_	Fe_2_O_3_	MnO	MgO	CaO	Na_2_O	K_2_O	P_2_O_5_
Content (%)	57.10	14.60	0.80	9.15	0.22	5.41	7.91	1.03	2.34	0.27

The reagents required for the experiment are as follows: FeSO_4_·7H_2_O, Na_2_S·9H_2_O, K_2_Cr_2_O_7_, Cu(NO_3_)_2_·3H_2_O, Cd(NO_3_)_2_·4H_2_O, NaOH, EDTA, sulfuric acid, phosphoric acid, nitric acid, diphenylcarbazide, acetone. The above reagents are all analytically pure and produced by National Pharmaceutical Group Chemical Reagents Co., Ltd (Shanghai, China). Deionized water was used throughout the experiment.

#### 2.1.2 Simulated AMD wastewater

Based on the measured data of AMD from a mining area in Yulin City, Shaanxi Province, a synthetic mixed water sample containing Cr(vi), Cu(ii), and Cd(ii) was prepared. The ion concentration of Cr(vi), Cu(ii) and Cd(ii) in the simulated AMD water sample was set to 100 mg L^−1^, and the pH of the solution was adjusted to 4 by titration with 0.1 mol per L H_2_SO_4_ and 0.1 mol per L NaOH. The simulated AMD contained Cr(vi), Cu(ii), and Cd(ii) as the primary metal ions, Additionally, trace metals such as Fe^3+^ and Al^3+^ (derived from coal gangue dissolution) were monitored but found at negligible levels (<1 mg L^−1^).

### 2.2 Experimental methods

#### 2.2.1 Preparation of nFeS-CG

Prepare 200 mL of Na_2_S solution with a concentration of 0.30 mol L^−1^ in a conical flask, add an appropriate amount of coal gangue, and stir with a magnetic stirrer for 6 h until S^2−^ reaches saturation on the coal gangue. After standing for 5 min, pour out the upper layer of solution, leaving only the particles at the bottom of the conical flask. Prepare 200 mL of 0.53 mol per L FeSO_4_ solution, load the conical flask containing coal gangue particles into the ultrasonic cleaner, drip FeSO_4_ solution into the conical flask through the peristaltic pump, adjust the drip flow rate to 26.4 mL min^−1^, and use a mechanical agitator to stir at 350 rpm. The whole ultrasonic environment is set to ultrasonic treatment at 40 kHz at room temperature for 10 min. The suspension after ultrasonic treatment was washed three times with deionized water, placed in a centrifuge, centrifuged at 1000 rpm for 15 min, vacuum dried, sealed and stored in a refrigerator at low temperature. The preparation process was repeated three times, and the products were mixed uniformly for subsequent experiments.

#### 2.2.2 Performance evaluation

In order to determine the application potential of nFeS-CG, the adsorption of Cr(vi), Cu(ii) and Cd(ii) in AMD was studied. The study examined how various factors—such as adsorbent dosage, initial pH, initial concentration, and reaction time—affect the removal of Cr(vi), Cu(ii), and Cd(ii) from AMD. Three parallel experiments were set up in each group, and the mean value was taken as the final measured value. The removal efficiency (*R*, %) and adsorption capacity (*q*, mg g^−1^) were calculated as follows:
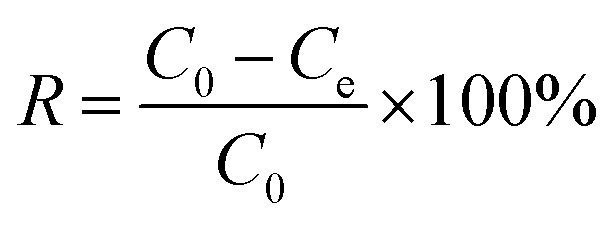

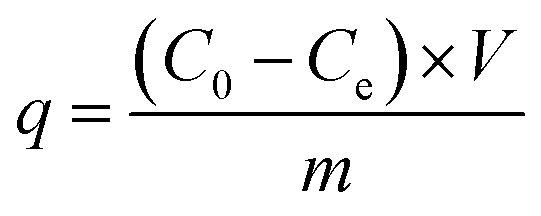
where: *R* is removal efficiency, %; *C*_0_ and *C*_e_ are the initial and residual concentrations of Cr(vi), Cu(ii), and Cd(ii), respectively, mg L^−1^. *V* is the volume of solution, L; *m* is the mass of adsorbent, g.

Experimental procedure: at 298.15 K, under the condition of an initial pH of 4 and an initial concentration of Cr(vi), Cu(ii) and Cd(ii) at 100 mg L^−1^, different masses (5, 6, 7, 8, 9 g L^−1^) of nFeS-CG were added to 200 mL of simulated AMD. The solution was stirred at a speed of 250 rpm for 155 min, then samples were taken and their concentrations were measured to investigate the effect of the adsorbent dosage on the removal of Cr(vi), Cu(ii) and Cd(ii) by nFeS-CG. Each dosage gradient was tested in triplicate, and the removal rates were calculated as the mean value of three parallel measurements to ensure reliability. At 298.15 K, under the condition of varying initial pH values (3, 4, 5, 6, 7) and initial concentrations of Cr(vi), Cu(ii) and Cd(ii) at 100 mg L^−1^, 7 g L^−1^ of nFeS-CG was added to 200 mL of simulated AMD. The solution was stirred at a speed of 250 rpm for 155 min, then samples were taken and their concentrations were measured to investigate the effect of the initial pH of the solution on the removal of Cr(vi), Cu(ii) and Cd(ii) by nFeS-CG. Triplicate experiments were performed for each pH value, and the average removal efficiency was used for subsequent analysis. At 298.15 K, under the condition of an initial pH of 4 and initial concentrations of Cr(vi), Cu(ii) and Cd(ii) at 100 mg L^−1^, 7 g L^−1^ of nFeS-CG was added to 200 mL of simulated AMD. The solution was stirred at a speed of 250 rpm for different contact times (5, 20, 35, 50, 65, 80, 95, 110, 125, 140, 155 min), then samples were taken and their concentrations were measured to investigate the effect of contact time on the removal of Cr(vi), Cu(ii) and Cd(ii) by nFeS-CG. For each contact time, three parallel experiments were conducted, and the mean removal rate was calculated to reduce experimental error. At 298.15 K, under the condition of an initial pH of 4, and varying initial concentrations of Cr(vi), Cu(ii) and Cd(ii) (50, 70, 100, 150, 200 mg L^−1^), 7 g L^−1^ of nFeS-CG was added to 200 mL of simulated AMD with different concentrations of Cr(vi), Cu(ii) and Cd(ii). The solution was stirred at a speed of 250 rpm for 155 min, then samples were taken and their concentrations were measured to investigate the effect of the initial concentrations of Cr(vi), Cu(ii) and Cd(ii) on the removal of Cr(vi), Cu(ii) and Cd(ii) by nFeS-CG. Each initial concentration was tested in triplicate, and the adsorption capacity was determined as the average of three parallel measurements.

#### 2.2.3 Adsorption kinetics and adsorption isotherms

(1) Adsorption kinetics test: take 200 mL of AMD wastewater with Cr(vi), Cu(ii), and Cd(ii) concentrations of 100 mg L^−1^, and set the wastewater pH to 4. Add 1.4 g of nFeS-CG adsorbent and stir with a magnetic stirrer at 250 rpm at room temperature. Measure the concentrations of Cr(vi), Cu(ii), and Cd(ii) in the water sample at reaction times of 5 min, 20 min, 35 min, 50 min, 65 min, 80 min, 95 min, 110 min, 125 min, 140 min, and 155 min. Three parallel experiments were set up in each group, and the mean value was taken as the final measured value. All time-point measurements were conducted in triplicate, and the mean adsorption capacity was recorded. The equation is as follows:

1 ln(*q*_e_ − *q*_*t*_) = ln *q*_e_ − *k*_1_*t*

can be transformed into:^22^

2 *q*_*t*_ = *q*_e_(1 − e^−*k*_1_*t*^)

pseudo-second order kinetics model:^23^3
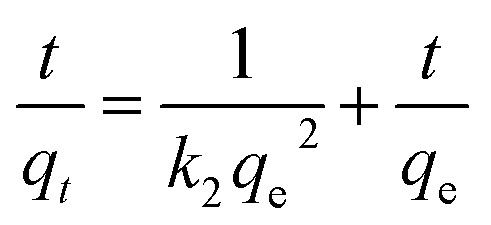

4 *q*_*t*_ = *k*_p_*t*^1/2^ + *C*

intraparticle diffusion model:

Elovich model:5
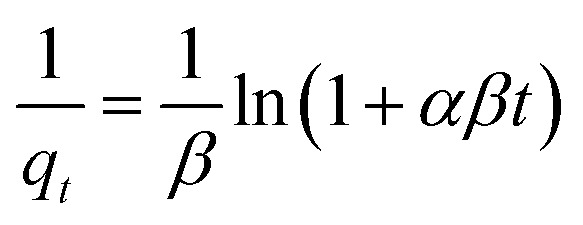
where: *q*_*t*_ is the adsorption capacity of the adsorbent for pollutants at time *t*, mg g^−1^*q*_e_ is the adsorption capacity of the adsorbent for pollutants at equilibrium, mg g^−1^; *k*_1_ is the pseudo-first-order adsorption rate constant, min; *k*_2_ is the pseudo-second-order adsorption rate constant, mg g^−1^; *k*_p_ is the intraparticle diffusion rate constant, mg g^−1^ min^−1/2^; *C* is the thickness of the boundary layer involved; *α* is the initial adsorption rate, mg g^−1^ min^−1/2^; *β* is the activation energy parameter of chemisorption on adsorbent surface, g mg^−1^.

(2) Adsorption isotherm test: the initial concentrations of Cr(vi), Cu(ii) and Cd(ii) in 200 mL AMD wastewater were 50 mg L^−1^, 70 mg L^−1^, 100 mg L^−1^, 150 mg L^−1^ and 200 mg L^−1^, respectively. The pH of the wastewater was set to 4. Add 7 g per L nFeS-CG adsorbent each time, stir with a magnetic stirrer at 250 rpm at room temperature, and detect the concentrations of Cr(vi), Cu(ii), and Cd(ii) in the water sample at a reaction time of 155 min. Three parallel experiments were set up in each group, and the mean value was taken as the final measured value. Each initial concentration gradient was tested in triplicate, and the equilibrium adsorption capacity was averaged. The equation is as follows:

Langmuir model:^24^6
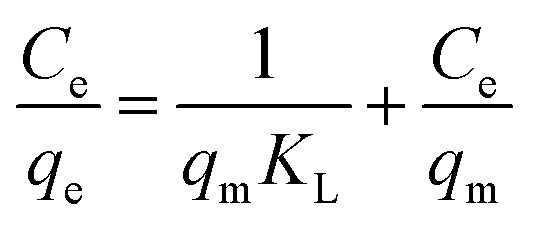


Freundlich model:

7 *q*_e_ = *K*_F_*C*_e_^1/*n*^

can be transformed into:^25^8
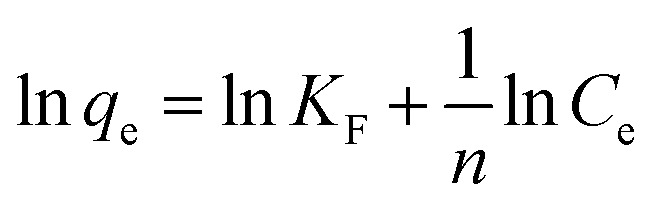


Temkin model:9
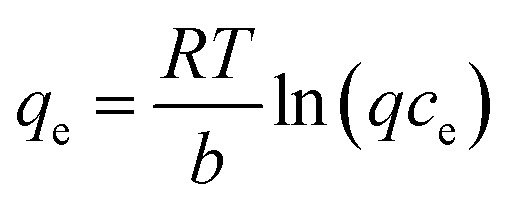
where: *C*_e_ is the pollutant concentration at solution equilibrium, mg L^−1^; *q*_e_ is the adsorption capacity of the adsorbent for pollutants at equilibrium, mg g^−1^; *q*_m_ is the saturated adsorption capacity of the adsorbent for pollutants, mg g^−1^; *K*_L_ is the adsorption constant of Langmuir model; *K*_F_ is the adsorption constant of Freundlich model; *n* is the correlation constant of adsorption strength; *a* and *b* are Temkin isothermal adsorption constants; *T* is the absolute temperature of adsorption, K.

#### 2.2.4 Adsorption thermodynamics

In order to study the thermal effect in the adsorption process, the parameters of adsorption enthalpy Δ*H*° (kJ mol^−1^), adsorption entropy Δ*S*° (J mol^−1^ K^−1^) and Gibbs free energy Δ*G*° (kJ mol^−1^) at different temperatures were calculated based on the experimental data of adsorption isotherms. The equation is as follows:^26^

10 Δ*G*° = −*RT* ln *K*_L_

11
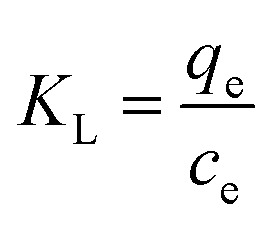

12 Δ*G*° = Δ*H*° − *T*Δ*S*°

eqn (11) can be transformed into:13
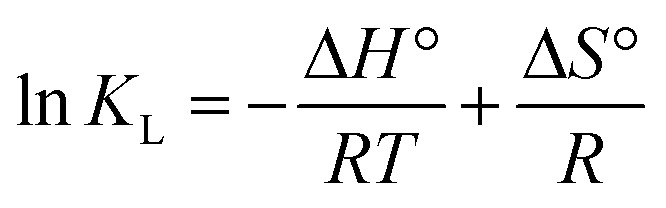
where: *T* is the thermodynamic temperature, K; *K*_L_ is the adsorption equilibrium coefficient; *R* is the ideal gas constant, mol K; *c*_e_ is the pollutant concentration at solution equilibrium, mg L^−1^; when *q*_e_ is equilibrium, the adsorption capacity of the adsorbent for pollutants, mg g^−1^. Triplicate experiments were performed at each temperature (298.15 K, 308.15 K, 318.15 K), and the thermodynamic parameters were calculated using average values.

#### 2.2.5 Coexisting ions

The effects of different coexisting ions on the simultaneous removal of Cr(vi), Cu(ii) and Cd(ii) by coal gangue loaded nano-FeS were investigated. In this study, the effects of coexisting cations of Zn(ii) and Ni(ii) on the simultaneous removal of Cr(vi), Cu(ii) and Cd(ii) by coal gangue loaded nano-FeS (nFeS-CG) were investigated. In the experiment, the concentration of Zn(ii) and Ni(ii) was 100 mg L^−1^, and the mixed solution was prepared with 100 mg L^−1^ Cr(vi), Cu(ii) and Cd(ii), respectively. At the same time, a blank control group without external ions was set up. Each group was added with 7 g per L nFeS-CG and stirred at 250 rpm for 155 min at room temperature to detect the target ion concentration in water samples. Three parallel experiments were set up in each group, and the mean value was taken as the final measured value.

#### 2.2.6 Leaching toxicity

According to the “solid waste leaching toxicity leaching method-sulfuric acid nitric acid method(HJ/T300-2007)”, sulfuric acid and nitric acid with a mass ratio of 2 : 1 were added to deionized water to make the solution pH 3.1–3.3. The dried coal gangue and nFeS-CG were added to the above extract at a solid–liquid ratio of 1 : 10 (g mL^−1^), and stirred at room temperature for 18 h. The concentration of main heavy metal ions was detected by flame atomic spectrophotometer. Three parallel experiments were set up in each group, and the mean value was taken as the final measured value.

#### 2.2.7 Regeneration

The reusability of nFeS-CG was evaluated by three cycles of adsorption–desorption experiments. In the experiment, 200 mL of simulated AMD wastewater was taken each time, and 7 g per L nFeS-CG adsorbent was added. The adsorption process was completed by stirring at 250 rpm for 155 min at room temperature. Subsequently, 0.5 mol per L EDTA eluent was used for desorption and regeneration treatment, stirring for 3 h. After desorption, the adsorbent was centrifuged, cleaned and dried to be reused for Cr(vi), Cu(ii) and Cd(ii) adsorption experiments under the same experimental conditions. Three parallel experiments were set up in each group, and the mean value was taken as the final measured value.

#### 2.2.8 Characterization and detection

Scanning electron microscopy (SEM, MERLIN Compact, Zeiss, Germany) was used to analyze the micro-morphology of coal gangue and nFeS-CG before and after treatment of simulated AMD. X-ray diffraction (XRD, D8 ADVANCE, Bruker, Germany) was used to analyze the mineral composition of coal gangue and nFeS-CG before and after AMD treatment. Fourier transform infrared spectroscopy (FTIR, Summit X, Thermo Fisher Scientific, USA) was used to analyze the changes of surface functional groups before and after the treatment of AMD by coal gangue and nFeS-CG. The morphology and size of nano-FeS on the surface of nFeS-CG were analyzed by transmission electron microscopy (TEM, Talos F200X G2, Semmerfeld, USA). X-ray photoelectron spectroscopy (XPS, Thermo Kalpha, China) was used to analyze the changes of elemental valence before and after the treatment of AMD by coal gangue and nFeS-CG.

Cr(vi) was measured using the diphenylcarbazide spectrophotometric method (GB/T7467-87), Cu(ii) and Cd(ii) were determined by flame atomic spectrophotometry (HJ757-2015, GB7475-87), and pH determination was carried out by the glass electrode method as per GB/T6920-86.

## 3. Results and discussion

### 3.1 Performance evaluation test

#### 3.1.1 Effect of adsorbent dosage

**Fig. 1:**
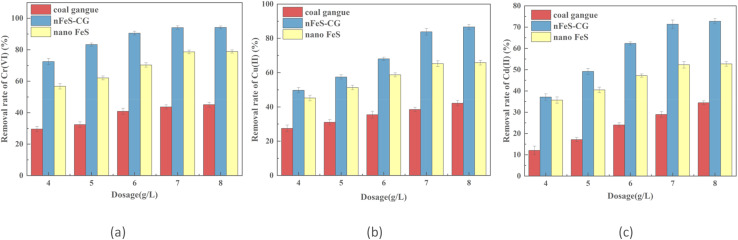
The effects of adsorbent dosage on removal of Cr(vi), Cu(ii) and Cd(ii). Error bars represent standard deviation of 3 repetitions.

It can be seen from Fig. 1 that the removal rates of Cr(vi), Cu(ii) and Cd(ii) by nFeS-CG increased rapidly with the increase of nFeS-CG dosage. The increase of dosage means the increase of adsorption sites, which promotes the collision between adsorbent and pollutants. When the dosage was 7.0 g L^−1^, the removal rates of Cr(vi), Cu(ii) and Cd(ii) by nFeS-CG reached the maximum, which were 94.08%, 83.83% and 71.36% respectively. Continue to increase the dosage of each ion removal rate did not increase significantly, indicating that the adsorption has reached equilibrium.^27^ The removal efficiency of Cr(vi), Cu(ii) and Cd(ii) by nFeS-CG was significantly higher than that of coal gangue, indicating that the loading of nano-FeS effectively improved the fixation ability of coal gangue to Cr(vi), Cu(ii) and Cd(ii). In addition, the comparative experiments showed that when FeS was used alone, the removal rates of Cr(vi), Cu(ii) and Cd(ii) at 7 g per L dosage were 78.6%, 65.2% and 52.3%, respectively, which were lower than those of nFeS-CG. This indicates that the coal gangue carrier significantly improves the adsorption activity of the material by inhibiting FeS agglomeration, and verifies the rationality of the composite material design. In summary, the dosage of 7.0 g L^−1^ was selected for subsequent experiments.

#### 3.1.2 Effect of initial pH

**Fig. 2:**
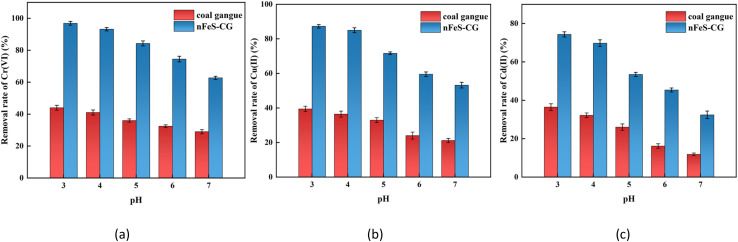
The effects of solution initial pH on removal of Cr(vi), Cu(ii) and Cd(ii). Error bars represent standard deviation of 3 repetitions.

From Fig. 2, it can be seen that with the increase of solution pH, the removal rate of Cr(vi), Cu(ii) and Cd(ii) by nFeS-CG gradually decreased. When pH = 3, the removal rates of Cr(vi), Cu(ii) and Cd(ii) were 96.90%, 87.26% and 74.33%, respectively. When pH = 7, the removal rates of Cr(vi), Cu(ii) and Cd(ii) were 62.70%, 53.19% and 32.41%, respectively. The removal rates of Cr(vi), Cu(ii) and Cd(ii) by nFeS-CG decreased with the increase of pH. The reason is that acidic conditions can promote the dissolution and ionization of FeS and accelerate the release of more Fe^2+^ and S^2−^, which further contributed to the overall reaction process.^28^ At the same time, under lower pH conditions, the surface of coal gangue is easily protonated by H^+^ and positively charged, which enhances the electrostatic attraction of nFeS-CG to Cr(vi) and accelerates the removal of Cr(vi). In addition, because the solubility product of Cu(ii) and Cd(ii) is smaller, it is easier to react with S^2−^ to form insoluble precipitates such as CuS and CdS, so that the removal rate of Cu(ii) and Cd(ii) is higher.^29^ With the increase of solution pH, the solubility of FeS decreased, and the removal rates of Cr(vi), Cu(ii) and Cd(ii) by nFeS-CG decreased gradually.

Under acidic conditions, the Fe^2+^ released by nFeS-CG preferentially undergoes an electron transfer reaction with Cr(vi). The kinetic rate of this reaction is much higher than that of the precipitation reaction of Cu(ii), Cd(ii) and S^2−^, resulting in the rapid removal of Cr(vi) in a short time. At low pH, the surface of coal gangue is positively charged due to protonation, which has a stronger electrostatic attraction with negatively charged Cr(vi), while Cu(ii) and Cd(ii) exist in the form of cations, which need to compete for limited S^2−^ to generate precipitation, so the removal efficiency is low. The Cr_2_S_3_ formed by Cr(iii) and S^2−^ is more stable than CuS and CdS, which further promotes the efficient removal of Cr(vi).

#### 3.1.3 Effect of reaction time

**Fig. 3:**
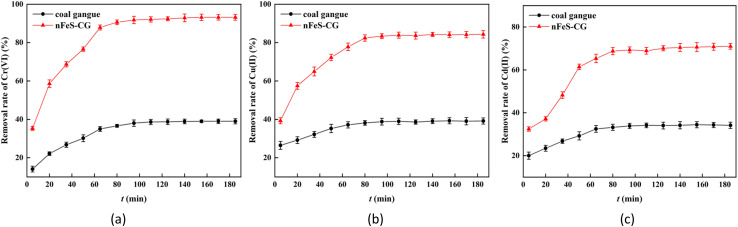
The effects of reaction time on removal of Cr(vi), Cu(ii) and Cd(ii). Error bars represent standard deviation of 3 repetitions.

From Fig. 3, it can be seen that with the increase of reaction time, the removal rate of Cr(vi), Cu(ii) and Cd(ii) by nFeS-CG increases rapidly and then tends to be stable. When the reaction time was 95 min, the removal rates of Cr(vi), Cu(ii) and Cd(ii) were 91.80%, 83.36% and 69.31%, respectively. In the early stage of the reaction, the removal rate of each ion was higher, because nFeS-CG provided a large number of adsorption sites for Cr(vi), Cu(ii) and Cd(ii), and the kinetic potential energy of the adsorption reaction was higher, so the removal rate increased faster. At the same time, it can be observed that the removal rate of Cr(vi) is slightly higher than that of other ions. The reason is that FeS undergoes rapid dissolution ionization under acidic conditions, and Fe^2+^ can quickly reduce Cr(vi) to Cr(iii). With the increase of pH, a large amount of sulfides and hydroxides precipitate on the surface of nFeS-CG. When the reaction time was 155 min, the removal rates of Cr(vi), Cu(ii) and Cd(ii) were 93.74%, 84.08% and 70.19%, respectively. The removal rate of each ion reached the maximum, and the removal rate of the continuous reaction did not change significantly, indicating that nFeS-CG had reached the adsorption saturation state.^30^ Considering comprehensively, the reaction time was set to 155 min as the reaction time of subsequent experiments.

#### 3.1.4 Effect of initial concentration

**Fig. 4:**
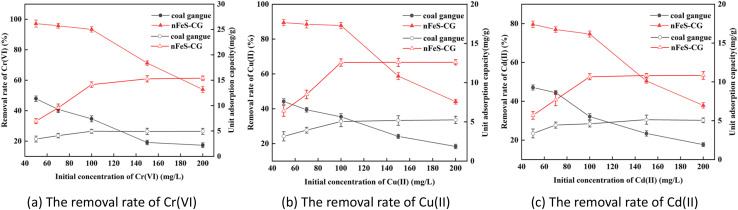
The effects of initial concentration on removal of Cr(vi), Cu(ii), and Cd(ii). Error bars represent standard deviation of 3 repetitions.

It can be seen from Fig. 4 that with the increase of the initial concentration of the solution, the removal rates of Cr(vi), Cu(ii) and Cd(ii) by nFeS-CG gradually decreased, and the unit adsorption capacity increased first and then gradually leveled off. When the concentration of Cr(vi) is 50 mg L^−1^, the unit adsorption capacity is 6.94 mg g^−1^. When the concentration of Cr(vi) is 100 mg L^−1^, the unit adsorption capacity can reach 14.14 mg g^−1^. Continue to increase the initial concentration, the unit adsorption capacity is basically unchanged. The removal rate of Cu(ii) decreased from 89.46% to 44.10%. When the initial concentration was 50–100 mg L^−1^, the unit adsorption capacity increased from 6.39 mg g^−1^ to 12.57 mg g^−1^. When the initial concentration was 100–200 mg L^−1^, the unit adsorption capacity increased slowly. The removal rate of Cd(ii) decreased from 79.63% to 37.94%. When the initial concentration was 50–100 mg L^−1^, the unit adsorption capacity increased from 5.68 mg g^−1^ to 10.66 mg g^−1^. When the initial concentration was 100–200 mg L^−1^, the unit adsorption capacity increased slowly. In the early stage of the reaction, nFeS-CG can fully react with each ion. With the increase of ion concentration, the adsorption site of nFeS-CG is insufficient, and the removal rate decreases.^31^ In addition, the precipitation formed by the reaction is attached to the surface of nFeS-CG, which is not conducive to the reaction. Comprehensive consideration, the initial concentration of Cr(vi), Cu(ii) and Cd(ii) in the subsequent experiment was set to 100 mg L^−1^.

### 3.2 Adsorption kinetics

**Fig. 5:**
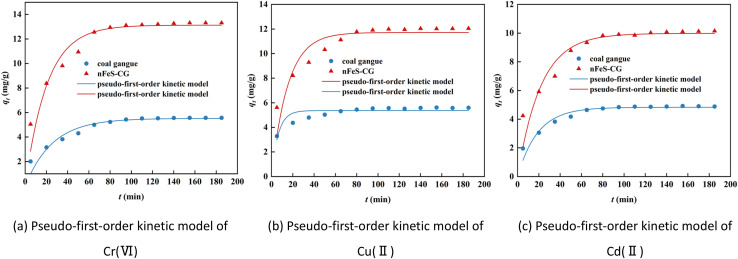
The pseudo-first-order kinetic model for the adsorption of Cr(vi), Cu(ii) and Cd(ii).

Fig. 5 shows the pseudo-first-order kinetic model fitting curves of Cr(vi), Cu(ii) and Cd(ii) adsorption on nFeS-CG. The relevant parameters are listed in Table 2. The adsorption of Cr(vi), Cu(ii) and Cd(ii) by nFeS-CG was different from the fitting degree of the pseudo-first-order kinetic model. The theoretical equilibrium adsorption capacity of each ion is quite different from the actual equilibrium adsorption capacity. Moreover, the quasi-first-order kinetic model is only suitable for describing the kinetic behavior in the early stage of adsorption, and cannot describe the whole process of adsorption.

**Table 2:** Adsorption kinetics parameters of Cr(vi), Cu(ii) and Cd(ii) by coal gangue and nFeS-CG

Model	Parameter	Cr(vi)		Cu(ii)		Cd(ii)	
		CG	nFeS-CG	CG	nFeS-CG	CG	nFeS-CG
Quasi-first-order kinetics	*k*_1_	0.0390	0.0488	0.1611	0.0656	0.0530	0.0459
	*q*_e_	5.5186	13.1263	5.3649	11.7173	4.8270	9.9790
	*R* ^2^	0.9029	0.9026	0.7206	0.8130	0.9020	0.8548
Pseudo-second order kinetics	*k*_2_	0.0104	0.0073	0.0418	0.0118	0.0180	0.0094
	*q*_e_	6.1381	14.1170	5.6998	12.4550	5.2376	10.7139
	*R* ^2^	0.9689	0.9863	0.9675	0.9745	0.9751	0.9724
Intra-particle diffusion	*k*_p1_	0.5629	1.3985	0.3931	0.9623	0.5548	0.7650
	*C*_1_	0.7631	2.3778	2.4594	3.5838	0.7968	2.6191
	*R* ^2^	0.8957	0.9033	0.9378	0.9518	0.9412	0.9544
	*k*_p2_	0.4234	0.7441	0.2240	0.7451	0.2814	0.9125
	*C*_2_	1.4637	6.0844	3.4251	4.9851	2.2685	1.8909
	*R* ^2^	0.9293	0.8891	0.9587	0.9083	0.9318	0.8899
	*k*_p3_	0.0318	0.0799	0.0152	0.0309	0.0265	0.0742
	*C*_3_	5.1631	12.2815	5.3905	11.6300	4.5598	9.1559
	*R* ^2^	0.7185	0.8667	0.3201	0.7255	0.5938	0.8845
Elovich	*α*	1.0616	3.9754	26.2960	7.8207	1.8036	3.0921
	*β*	0.8744	0.4029	1.5343	0.5238	1.1428	0.5366
	*R* ^2^	0.9593	0.9471	0.9541	0.9547	0.9418	0.9336

**Fig. 6:**
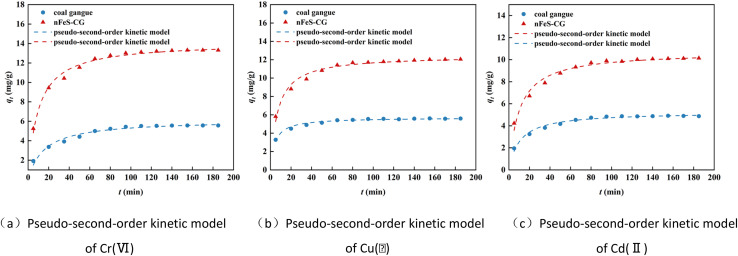
The pseudo-second-order kinetic model for the adsorption of Cr(vi), total chromium, Cu(ii) and Cd(ii).

Fig. 6 shows the pseudo-second-order kinetic model fitting curves of Cr(vi), Cu(ii) and Cd(ii) adsorption on nFeS-CG. The relevant parameters are listed in Table 2. The adsorption of Cr(vi), Cu(ii) and Cd(ii) by nFeS-CG had a good correlation with the pseudo-second-order kinetic model (*R*^2^ > 0.95). The theoretical equilibrium adsorption capacity of each ion is very close to the actual equilibrium adsorption capacity. It indicated that the adsorption of Cr(vi), Cu(ii) and Cd(ii) by nFeS-CG was mainly chemical adsorption.^22^ Since the pseudo-second-order kinetic model includes all adsorption processes such as surface adsorption, internal diffusion and external diffusion, both physical adsorption and chemical adsorption exist. Therefore, the pseudo-second-order kinetic can better reflect the removal mechanism of Cr(vi), Cu(ii) and Cd(ii) by nFeS-CG. In order to determine the diffusion mechanism of each ion on nFeS-CG, the intra-particle diffusion and Elovich model were used to fit the adsorption process.

**Fig. 7:**
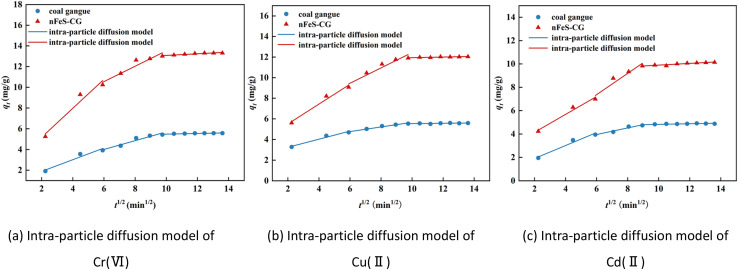
Intraparticle diffusion model for the adsorption of Cr(vi), Cu(ii) and Cd(ii).

**Fig. 8:**
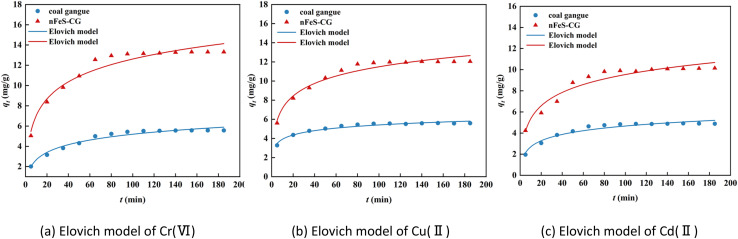
Elovich model for the adsorption of Cr(vi), Cu(ii) and Cd(ii).

It can be seen from Fig. 7 and Table 2 that the fitting curves of each ion intraparticle diffusion model are not at the origin, and the parameter *C* is not 0, indicating that intraparticle diffusion is not the only control step. The adsorption of metal ions by nFeS-CG can be divided into three stages, indicating that the adsorption is a continuous segmented process.^23^ The first stage is surface adsorption. At the initial stage of the reaction, the adsorption sites on the adsorbent are sufficient, and the dynamic potential energy difference between the metal ions and the nFeS-CG is large, which increases the probability of contact between the metal ions and the adsorption sites, and the adsorption rate is faster. The second stage is intraparticle diffusion. At this stage, the concentration of metal ions in the solution is low, the available adsorption sites on the surface of nFeS-CG are reduced, and the mass transfer power is insufficient, resulting in a decrease in the adsorption rate. The third stage is the adsorption equilibrium stage, in which the adsorption rate further slows down until equilibrium is reached. Among them, the correlation coefficient *R*^2^ of the first two stages is higher, indicating that surface diffusion and pore diffusion are the main rate-limiting steps in the adsorption of Cr(vi), Cu(ii) and Cd(ii) by nFeS-CG. In addition, the correlation coefficient *R*^2^ of the Elovich model for the adsorption of Cr(vi), Cu(ii) and Cd(ii) by nFeS-CG was greater than 0.92, indicating that chemical adsorption was the main rate-limiting step in the adsorption process. Therefore, the adsorption of metal ions by nFeS-CG is the result of chemical adsorption and multiple mechanisms (Fig. 8).

### 3.3 Adsorption isotherm

**Fig. 9:**
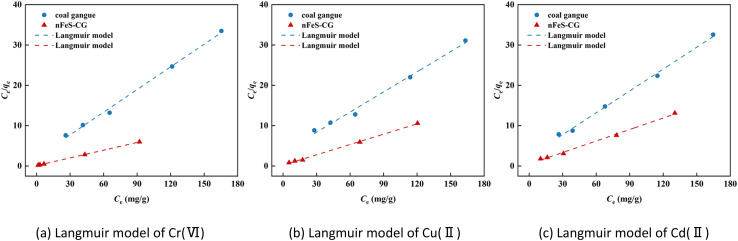
The Langmuir model fitting diagram of Cr(vi), Cu(ii) and Cd(ii).

**Fig. 10:**
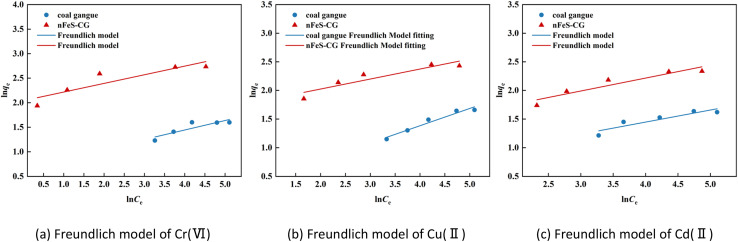
The Freundlich model fitting diagram of Cr(vi), Cu(ii) and Cd(ii).

**Fig. 11:**
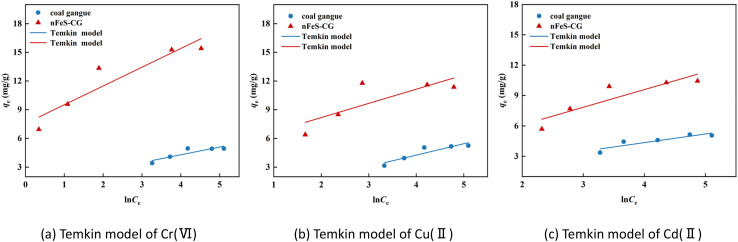
The Temkin model fitting diagram of Cr(vi), Cu(ii) and Cd(ii).

The adsorption behavior of Cr(vi), Cu(ii) and Cd(ii) by nFeS-CG and coal gangue at room temperature was fitted by Langmuir model, Freundlich model and Temkin model. The fitting curves are shown in Fig. 9–11. The relevant parameters are listed in Table 3.

**Table 3:** Adsorption isotherm parameters of Cr(vi), Cu(ii) and Cd(ii) by coal gangue nFeS-CG

Model	Parameter	Cr(vi)		Cu(ii)		Cd(ii)	
		CG	nFeS-CG	CG	nFeS-CG	CG	nFeS-CG
Langmuir	*q*_m_	5.34	15.70	6.01	12.61	5.54	10.52
	*K*_L_	0.0887	0.6421	0.0493	0.3886	0.0770	0.1925
	*R* ^2^	0.9945	0.9999	0.9887	0.9976	0.9947	0.9949
Freundlich	*n*	3.5244	6.4487	3.3240	5.7166	4.7549	4.4209
	*K*_F_	1.3187	5.8684	1.1979	5.3358	1.8329	3.7103
	*R* ^2^	0.7312	0.8737	0.9409	0.8158	0.7986	0.8568
Temkin	*K*_T_	3.6545	46.8488	0.6645	33.6203	2.9984	4.4047
	*B*	0.8100	1.9632	1.1862	1.4830	0.8531	1.7483
	*R* ^2^	0.7202	0.8290	0.7896	0.5348	0.7575	0.7497

It can be seen from Fig. 9 that the equilibrium adsorption capacity of nFeS-CG and coal gangue for Cr(vi), Cu(ii) and Cd(ii) increases with the increase of the initial concentration of each metal ion, indicating that high concentration is beneficial to the collision between adsorbate and adsorbent. It can be seen from Table 3 that the Langmuir model had the best fitting effect on the adsorption of Cr(vi), Cu(ii) and Cd(ii) by nFeS-CG and coal gangue (*R*^2^ = 0.9999, *R*^2^ = 0.9976, *R*^2^ = 0.9949; *R*^2^ = 0.9945, *R*^2^ = 0.9887, *R*^2^ = 0.9947), better than the other two adsorption models. At the same time, the theoretical maximum adsorption capacity (*q*_m_) of nFeS-CG and coal gangue obtained by Langmuir model fitting is close to the experimental equilibrium adsorption capacity (*q*_e_), indicating that the adsorbent has monolayer adsorption with each metal ion.^24^ The correlation coefficient *n* of adsorption strength of Freundlich model is greater than 1, indicating that the adsorption process is spontaneous.^25^ The greater the value of *K*_F_, the greater the adsorption capacity and adsorption strength. By comparing the *K*_F_ value, it can be seen that the *K*_F_ value of nFeS-CG is 4.5497, 4.1379 and 1.8771 higher than that of coal gangue, respectively, indicating that nFeS-CG can effectively improve the ability to treat Cr(vi), Cu(ii) and Cd(ii) in AMD.

### 3.4 Adsorption thermodynamics

**Fig. 12:**
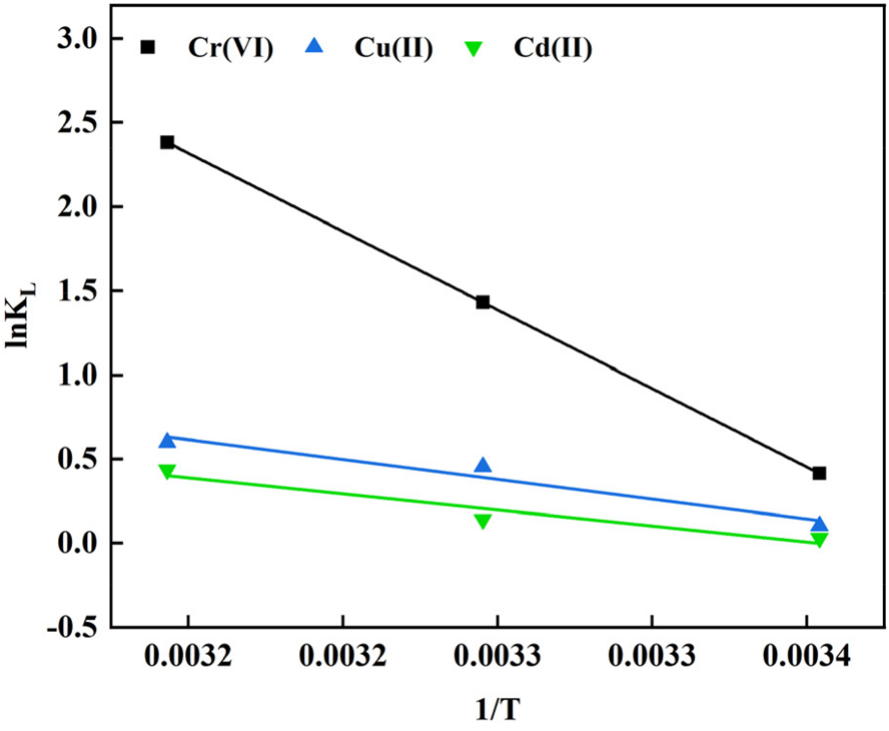
The Van't Hoff diagram for adsorption of Cr(vi), Cu(ii) and Cd(ii) by nFeS-CG.

Using Cr(vi), Cu(ii) and Cd(ii) as pollutants, the adsorption thermodynamic behavior of nFeS-CG on heavy metal ions at 298.15 K, 308.15 K and 318.15 K was investigated. The thermodynamic parameters are listed in Table 4. Fig. 12 is the van't Hoff diagram of nFeS-CG adsorbing Cr(vi), Cu(ii) and Cd(ii). The comparison of nFeS-CG with other reported adsorbents is shown in Table 5.

**Table 4:** Thermodynamic parameters of adsorption of Cr(vi), Cu(ii) and Cd(ii) by nFeS-CG

Ion	*T* (K)	Δ*G*° (kJ mol^−1^)	Δ*H*° (kJ mol^−1^)	Δ*S*° (J mol^−1^ K^−1^)
Cr(vi)	298.15	−1.03	77.63	263.83
	308.15	−3.67		
	318.15	−6.31		
Cu(ii)	298.15	−0.25	19.64	66.98
	308.15	−1.17		
	318.15	−1.58		
Cd(ii)	298.15	−0.07	15.90	53.31
	308.15	−0.36		
	318.15	−1.15		

**Table 5:** Comparison of properties of nFeS-CG and other adsorbents

Absorbent	*q*_m_ (mg g^−1^)			pH	Dosage (g L^−1^)	Equilibrium time	Temperature	Reference
	Cr(vi)	Cu(ii)	Cd(ii)					
nFeS-CG	15.70	12.61	10.52	4	7	155 min	25 °C	This study
Mg and Fe-LDHs AC		2.03		5	2.5	90 min	20 °C	33
Y molecular sieve loaded nano Fe^0^	13.20			5.2	1	120 min	25 °C	34
Ca and Al modified CG	9.19			5	10	100 min	20 °C	35
CG loaded chitosan	7.38			7	20	60 min	25 °C	36
Mechanochemical modified CG		5.69	6.73	6	1.67	120 min	20 °C	37

It can be seen from Table 4 that the Gibbs free energy Δ*G*° of Cr(vi), Cu(ii) and Cd(ii) adsorbed by nFeS-CG at different temperatures is less than 0, indicating that the adsorption process can proceed spontaneously.^26^ With the increase of temperature, the absolute value of Δ*G* also increases, indicating that the increase of temperature is beneficial to the adsorption process. The Δ*H* values of Cr(vi), Cu(ii) and Cd(ii) adsorbed by nFeS-CG were 77.63 kJ mol^−1^, 19.64 kJ mol^−1^ and 15.90 kJ mol^−1^, respectively, indicating that the adsorption of each metal ion by nFeS-CG was an endothermic process, and the increase of temperature was beneficial to the reaction. The entropy changes Δ*S*° were 263.83 J mol^−1^ K^−1^, 66.98 J mol^−1^ K^−1^ and 53.31 J mol^−1^ K^−1^, respectively, which were all positive values, indicating that the randomness of the whole adsorption system increased during the reaction.^32^ It can be seen from Table 5 that nFeS-CG has better adsorption capacity for Cr(vi), Cu(ii) and Cd(ii) than other adsorbents in the literature.

### 3.5 Experimental study on coexisting ions

**Fig. 13:**
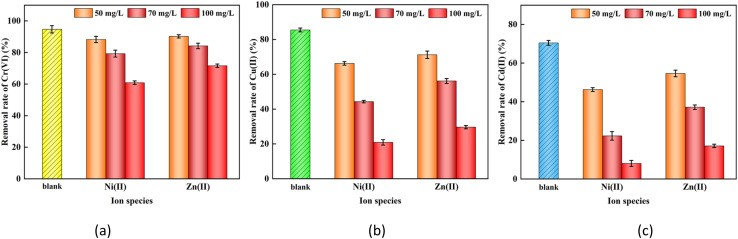
The removal efficiency of Cr(vi), Cu(ii) and Cd(ii) by nFeS-CG in the presence of Ni(ii) and Zn(ii). Error bars represent standard deviation of 3 repetitions.

In actual heavy metal wastewater, multiple ions often coexist, which may affect the removal of target pollutants by nFeS-CG. Therefore, in this study, the effects of coexisting ions and ionic strength on the removal of Cr(vi), Cu(ii) and Cd(ii) were investigated by using Ni(ii) and Zn(ii). The removal rates of each ion are shown in Fig. 13.

There was a significant competitive adsorption behavior among Cr(vi), Cu(ii) and Cd(ii). Under the same initial concentration and reaction conditions, the removal efficiency of nFeS-CG showed the rule of Cr(vi) > Cu(ii) > Cd(ii). This is due to three aspects: first, Cr(vi) exists in the form of anions such as HCrO_4_^−^ under acidic conditions, and forms a strong electrostatic attraction with the protonated nFeS-CG surface (positively charged), preferentially occupying the adsorption site, while Cu(ii) and Cd(ii) are cations, which need to compete with H^+^ and other cations for negative charge sites; Secondly, CuS (*K*_sp_ = 6.3 × 10^−36^) formed by Cu(ii) and S^2−^ is more stable than CdS (*K*_sp_ = 8 × 10^−27^). When S^2−^ is limited, Cu(ii) preferentially precipitates. Third, the hydrated ion radius of Cu(ii) is smaller than that of Cd(ii), and it is easier to diffuse to the surface of nFeS-CG and combine with functional groups to enhance the competitive advantage.

It can be seen from Fig. 13 that Ni(ii) and Zn(ii) have an inhibitory effect on the removal of Cr(vi), Cu(ii) and Cd(ii), and the inhibitory effect is proportional to the ionic strength. When the concentration of Ni(ii) was 100 mg L^−1^, the removal rates of Cr(vi), Cu(ii) and Cd(ii) in the system decreased by 33.82%, 64.57% and 62.43%, respectively. When the concentration of Zn(ii) was 100 mg L^−1^, the removal rates of Cr(vi), Cu(ii) and Cd(ii) in the system decreased by 23.65%, 55.83% and 53.33%, respectively. This is because the coexisting cations compete with Cr(vi), Cr(iii), Cu(ii) and Cd(ii) for adsorption sites, which weakens the electrostatic attraction between nFeS-CG and the target metal ions, thereby reducing the removal efficiency. At the same time, high ionic strength promoted the agglomeration of nano-FeS, reduced the specific surface area and strengthened its inhibition effect. In addition, it can be found that the competitive adsorption of Ni(ii) on Cr(vi), Cu(ii) and Cd(ii) systems is higher than that of Zn(ii), which may be attributed to the smaller ionic radius and higher electronegativity of Ni(ii).^38^ The influence of Ni(ii) and Zn(ii) on the adsorption of Cr(vi), Cu(ii) and Cd(ii) by nFeS-CG is: Ni(ii) > Zn(ii).

The competitive inhibitory effect of Ni(ii) on the target ions is stronger than that of Zn(ii), mainly due to the following differences in properties: the ionic radius of Ni^2+^(69 pm) is smaller than that of Zn^2+^(74 pm), and its charge density is higher. As a result, Ni^2+^ has a stronger electrostatic attraction to the negatively charged sites on the surface of nFeS-CG, making it more likely to occupy the adsorption sites. The solubility product (*K*_sp_) of NiS is 3.2 × 10^−19^, which is much smaller than that of ZnS (*K*_sp_ = 2.9 × 10^−25^). Under conditions where S^2−^ is limited, Ni^2+^ has a stronger binding affinity with S^2−^, preferentially forming stable precipitates. This reduces the amount of S^2−^ available for reactions with Cu(ii) and Cd(ii). The standard electrode potential of Ni^2+^(−0.25 V) is higher than that of Zn^2+^(−0.76 V). Ni^2+^ is more likely to undergo electron transfer with the reducing groups on the surface of nFeS-CG (such as Fe^2+^), indirectly inhibiting the reduction reaction of Cr(vi). These differences result in a more significant competitive interference of Ni(ii) on the target ions, which is consistent with the competitive behavior of heavy metal ions in actual AMD.

It is worth noting that the removal rate of Cd(ii) is most significantly inhibited by Ni(ii) and Zn(ii), which is closely related to the following factors: Cd(ii), Ni(ii) and Zn(ii) are divalent cations, which compete for the negative charge sites on the surface of nFeS-CG under acidic conditions. Due to the smaller hydrated ion radius and higher electronegativity of Ni(ii) and Zn(ii), their coordination ability with the adsorption sites is stronger, and the active sites are preferentially occupied, resulting in the significant inhibition of Cd(ii) adsorption. The solubility product of CdS is higher than that of ZnS and NiS. When S^2−^ is limited, Ni^2+^ and Zn^2+^ are more likely to combine with S^2−^ to form more stable sulfides, resulting in a decrease in S^2−^ involved in Cd(ii) precipitation, and the removal rate decreases more significantly. In addition, the stability of the inner complex formed by Fe oxides on the surface of nFeS-CG with Ni(ii) and Zn(ii) is higher than that with Cd(ii), which further reduces the binding efficiency of Cd(ii). This result indicates that the removal of Cd(ii) is more sensitive to coexisting cations in AMD containing multiple heavy metals, and pretreatment or adjustment of process parameters should be considered in practical applications to optimize its removal effect.

### 3.6 Leaching toxicity

The safety of adsorption materials in water treatment was evaluated by exploring the leaching toxicity of coal gangue and nFeS-CG. The content of heavy metals in leachate was determined by flame atomic spectrophotometer with reference to Chinese national standards (GB 5085.3-2007) and HJ/T299-2007.

The contents of main heavy metals in coal gangue and nFeS-CG leaching solution are shown in Table 6, including Cr, Pb, Cu, Cd, Zn and Ni. It can be seen from the table that the leaching concentration of nFeS-CG is lower than the limit standard value of leaching toxicity (GB 5085.3-2007), and is much lower than the leaching concentration of coal gangue, indicating that nFeS-CG has excellent immobilization ability for heavy metal ions. Under acidic conditions, the FeS crystal on the surface of nFeS-CG can quickly ionize a large amount of S^2−^, and the leaching heavy metal ions are fixed on nFeS-CG in the form of sulfide precipitation, which improves its safety in water treatment process. It can be used as an excellent adsorbent for treating wastewater containing Cr(vi), Cr(iii), Cu(ii) and Cd(ii).

**Table 6:** Main Heavy metal contents in coal gangue and nFeS-CG leachate (mg L^−1^)

Element	Mean value		Leaching toxicity standard value
	Coal gangue	nFeS-CG	
Cr	0.686	0.384	15
Pb	0.742	0.167	5
Cu	0.496	0.227	100
Cd	0.106	ND	1
Zn	0.283	0.149	100
Ni	0.079	ND	5

### 3.7 Experimental study on cyclic regeneration

**Fig. 14:**
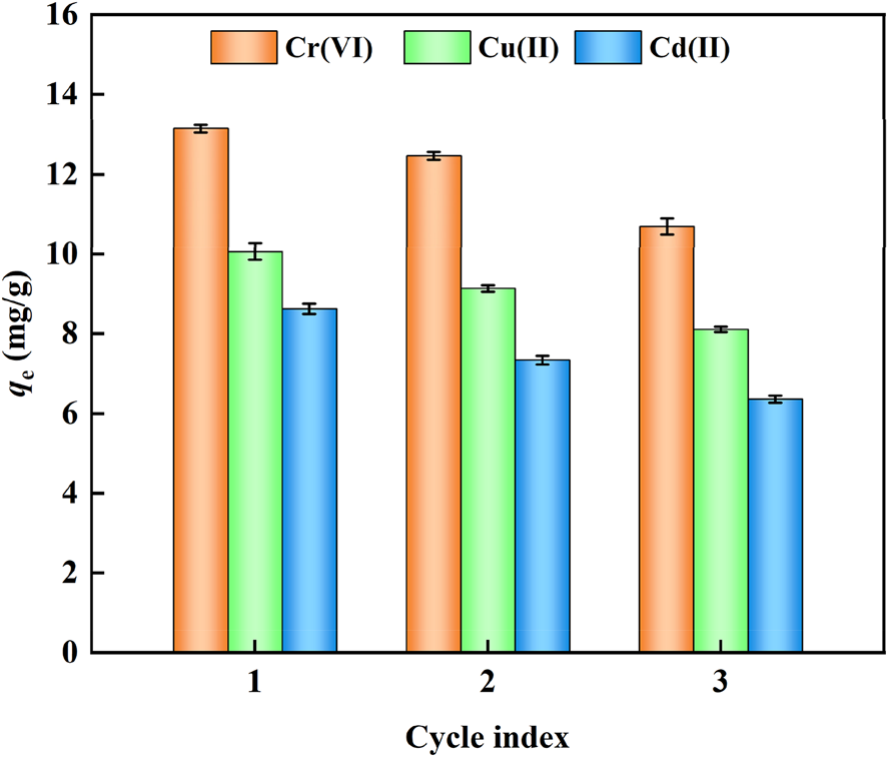
Recycling experiment of adsorption of Cr(vi), Cu(ii) and Cd(ii) by nFeS-CG. Error bars represent standard deviation of 3 repetitions.

The regeneration and reuse performance of nFeS-CG were further studied by desorption and regeneration experiments to prove the practicability and economy of the adsorbent in actual wastewater treatment. As shown in Fig. 14, with the increase of the number of cycles, the removal rate of each metal ion decreased to varying degrees, indicating that the loss of some reducing substances may occur during the regeneration process, resulting in the decrease of FeS content and adsorption sites. After three adsorption–desorption cycles, the adsorption capacities of nFeS-CG for Cr(vi), Cu(ii) and Cd(ii) were 10.69, 8.1 and 6.36 mg g^−1^, respectively, indicating that nFeS-CG had good regeneration performance.

### 3.8 Characterization analysis

#### 3.8.1 XRD analysis

**Fig. 15:**
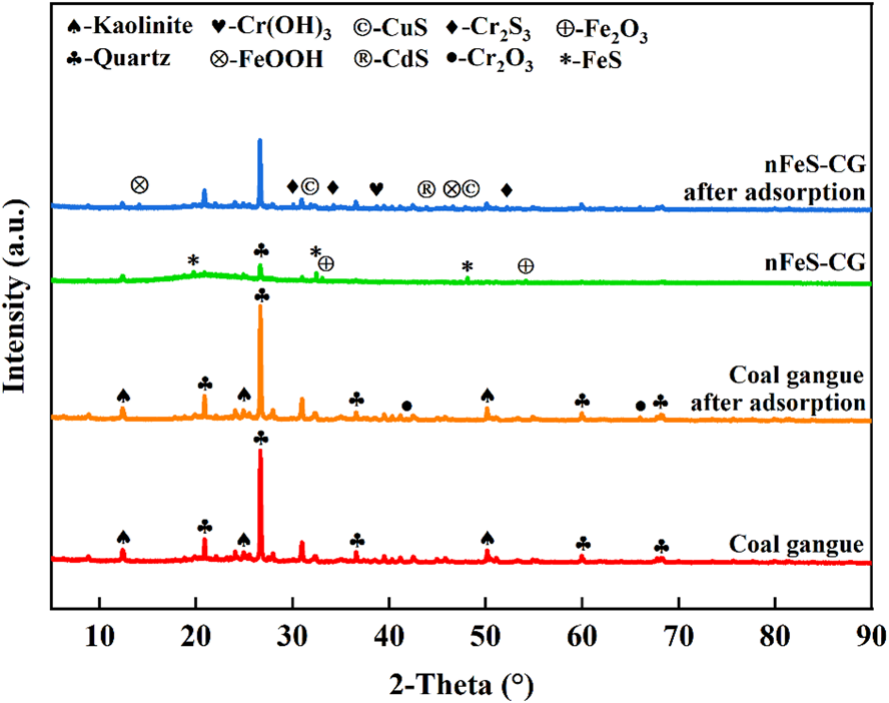
X-ray diffraction before and after the reaction of coal gangue with nFeS-CG.

The phase changes of coal gangue and nFeS-CG composites before and after adsorption were determined by XRD characterization technology, and the removal mechanism of Cr(vi), Cu(ii) and Cd(ii) by materials was analyzed and clarified. The X-ray diffraction patterns of coal gangue and nFeS-CG before and after reaction are shown in Fig. 15.

It can be seen from Fig. 15 that the characteristic diffraction peaks of kaolinite (PDF: #80-0886) appear at 2*θ* = 12.41°, 24.95° and 50.18°. The characteristic diffraction peaks of quartz (PDF: #86-1630) appeared at 2*θ* = 20.89°, 26.66°, 36.57°, 59.96° and 68.30°. The characteristic peaks of kaolinite and quartz in coal gangue after reaction are weakened to a certain extent. Because kaolinite, quartz and other crystals have strong acid resistance, they are not easy to be dissolved.^39^ The diffraction peaks of Cr_2_O_3_ (PDF: #84-0314) appear at the 2*θ* angles of 41.81° and 69.45° after the reaction of coal gangue, indicating that the oxygen-containing groups in coal gangue react with Cr(vi) to form Cr(iii), which is fixed on its surface in the form of Cr_2_O_3_ precipitate. The original characteristic diffraction peaks of coal gangue after loading modification all exist, and FeS diffraction peaks appear at 2*θ* = 19.76°, 32.45° and 48.11° (PDF: #76-0963), indicating that FeS is successfully loaded on the surface of coal gangue. The characteristic diffraction peaks of FeOOH (PDF: #73-2326) appeared at 2*θ* angles of 14.11° and 46.63°, and the weak characteristic diffraction peaks of Fe_2_O_3_ (PDF: #73-2234) appeared at 2*θ* angles of 33.08° and 54.17°, indicating that FeS has a weak oxidation.^40^ It can be seen from the diffraction pattern of nFeS-CG after adsorption that the characteristic peak of FeS disappeared, and new diffraction peaks appeared at 2*θ* angles of 30.07°, 34.23° and 52.18°, corresponding to the characteristic diffraction peaks of Cr_2_S_3_ (PDF: #72-1224). It shows that the supported nano-FeS has a reduction reaction with Cr(vi) in the dynamic experiment, and S^2−^ combines with Cr(iii) to form Cr_2_S_3_ precipitate.^41^ New diffraction peaks appear at 2*θ* angles of 31.87° and 47.89°, corresponding to the characteristic diffraction peaks of CuS (PDF: #75-2233), indicating that the hydrolysis product S^2−^ of the loaded nano-FeS reacts with Cu(ii). Similarly, a new diffraction peak appeared at 2*θ* angle of 43.82°, corresponding to the characteristic diffraction peak of CdS (PDF: #75-1546), indicating that the loaded nano-FeS reacted with Cd(ii) in the dynamic experiment.^42^ The characteristic diffraction peak of Cr(OH)_3_ (PDF: #16-0817) appeared at the 2*θ* angle of 38.71°, indicating that some Cr(iii) would be fixed to nFeS-CG in the form of Cr(OH)_3_ precipitate as the pH of the reaction system increased.

#### 3.8.2 SEM analysis

**Fig. 16:**
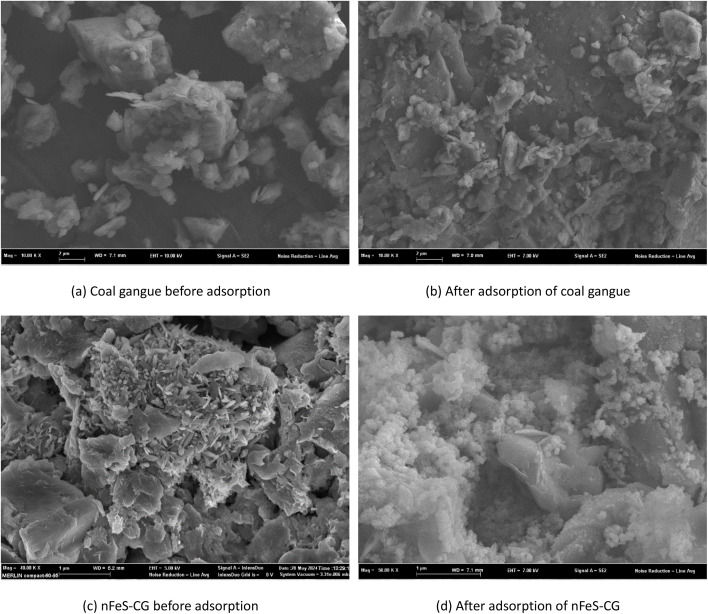
Scanning electron microscopy before and after the reaction of coal gangue with nFeS-CG.

The microstructure of coal gangue and nFeS-CG composites before and after adsorption was observed by SEM. Fig. 16(a–d) are the scanning electron micrographs of coal gangue before adsorption, coal gangue after adsorption, nFeS-CG before adsorption and nFeS-CG after adsorption, respectively.

Fig. 16(a) shows that the surface particles of coal gangue are loose, the size of layered structure is different and there are abundant pores.^43^ Comparing Fig. 16(a) and (b), it can be seen that the surface roughness of the adsorbed coal gangue increases and fine sediments appear. Combined with XRD analysis, it can be seen that the sediments are Cr_2_O_3_ precipitation and hydrolysis products of Cu(ii) and Cd(ii). Comparing Fig. 16(c) and (d), it can be seen that there are a large number of well-dispersed nano-FeS strip crystals on the surface of nFeS-CG before adsorption. It shows that the original structure of coal gangue has not been destroyed. After the reaction of nFeS-CG, the nano-FeS crystals loaded on nFeS-CG decreased, and a large number of particle precipitates appeared on the surface of coal gangue. XRD analysis showed that the particles were Cr_2_S_3_, CuS, CdS and Cr(OH)_3_ precipitates formed by the reaction of nano-FeS with Cr(vi), Cr(iii), Cu(ii) and Cd(ii).

#### 3.8.3 FTIR analysis

**Fig. 17:**
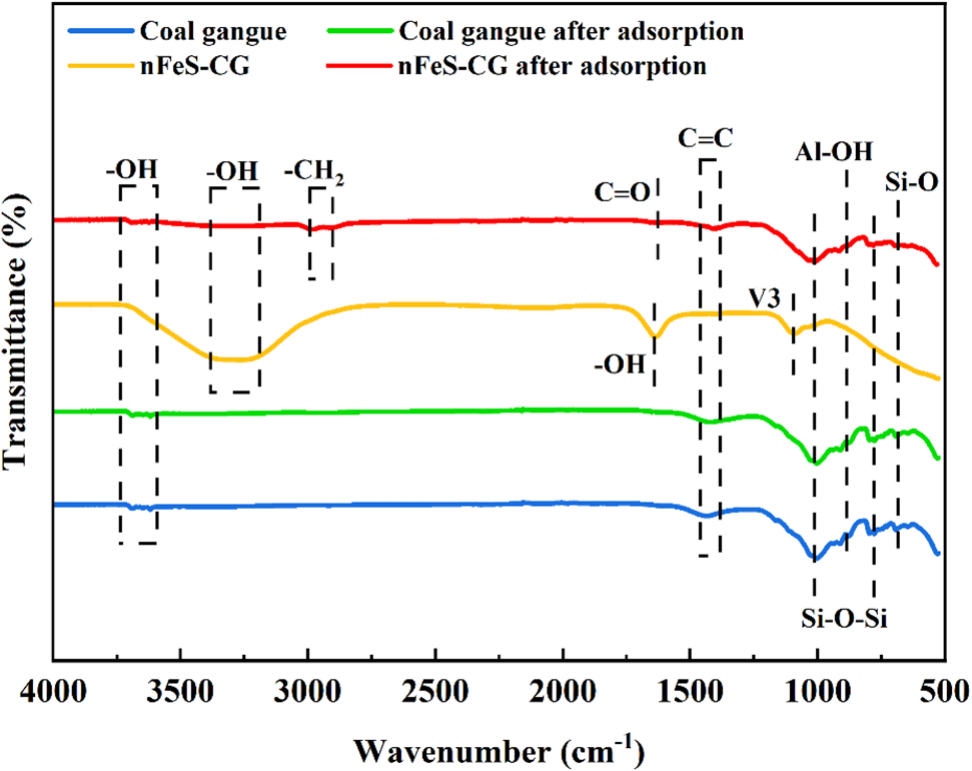
X-ray diffraction before and after the reaction of coal gangue with nFeS-CG.

The changes of functional groups before and after adsorption of coal gangue and nFeS-CG were analyzed by FTIR. Fig. 17 shows the infrared spectra of coal gangue and nFeS-CG before and after adsorption.

From the diagram, it can be seen that the functional groups of coal gangue before and after adsorption do not change significantly. There are similar characteristic peaks at 3682 cm^−1^ and 3617 cm^−1^, which correspond to the hydroxyl stretching vibration peak on the surface of coal gangue. The characteristic peaks at 1008 cm^−1^ and 784 cm^−1^ are the bending vibration peaks of Si–O–Si. 882 cm^−1^ and 684 cm^−1^ correspond to the bending vibration peaks of Al–OH and Si–O, respectively.^44^ At pH 3–4, the surface of coal gangue is rich in Al–OH groups, which are protonated under acidic conditions, rendering nFeS-CG positively charged. This facilitates strong electrostatic attraction to negatively charged HCrO_4_^−^, explaining the higher Cr(vi) removal efficiency. As pH increases, the degree of protonation decreases, reducing the positive surface charge and weakening electrostatic attraction to HCrO_4_^−^, consistent with the observed decline in Cr(vi) removal. For Cu(ii) and Cd(ii), though present as cations, their removal is dominated by precipitation with S^2−^ released from nFeS under acidic conditions. The decreased removal efficiency with increasing pH further confirms that electrostatic adsorption plays a secondary role, synergizing with precipitation. After adsorption, the intensity of hydroxyl peaks at 3682 cm^−1^ and 3617 cm^−1^ decreases significantly, which, combined with the weakened signal of O 1s at 532.0 eV in XPS, indicates that these hydroxyl groups participate in coordination with Cr(iii) and Cu(ii). Additionally, the disappearance of the Al–OH characteristic peak at 882 cm^−1^ suggests chelation with Cd(ii), providing additional binding sites to assist heavy metal immobilization. In the nFeS-CG spectrum, the characteristic peaks at 1008 cm^−1^, 882 cm^−1^, 784 cm^−1^ and 684 cm^−1^ were weakened, and the characteristic diffraction peak of Na_2_SO_4_ (V3) appeared at 1096 cm^−1^, indicating that there was a certain oxidation of S^2−^. 1640 cm^−1^ corresponds to the bending vibration peak of H–O–H, and a strong and wide absorption peak appears in the range of 3395–3214 cm^−1^, which is caused by the stretching vibration of carboxylic acid and phenolic hydroxyl.^45^ After the reaction of nFeS-CG with metal ions, the stretching vibration peak of –CH_2_ appeared in the range of 2900–3000 cm^−1^. The absorption peaks at 3682 cm^−1^ and 3617 cm^−1^ disappeared, indicating that the hydroxyl group reacted with metal ions. Combined with the XRD spectrum, it can be seen that Cr(OH)_3_, FeOOH and other precipitates were formed. The stretching vibration peak of –C=O appeared at 1634 cm^−1^, and the absorption peak intensity of –C=C at 1406 cm^−1^ was enhanced, indicating that the reducing groups in coal gangue reacted with metal ions and were oxidized to –C=O and –C=C groups.

#### 3.8.4 XPS analysis

**Fig. 18:**
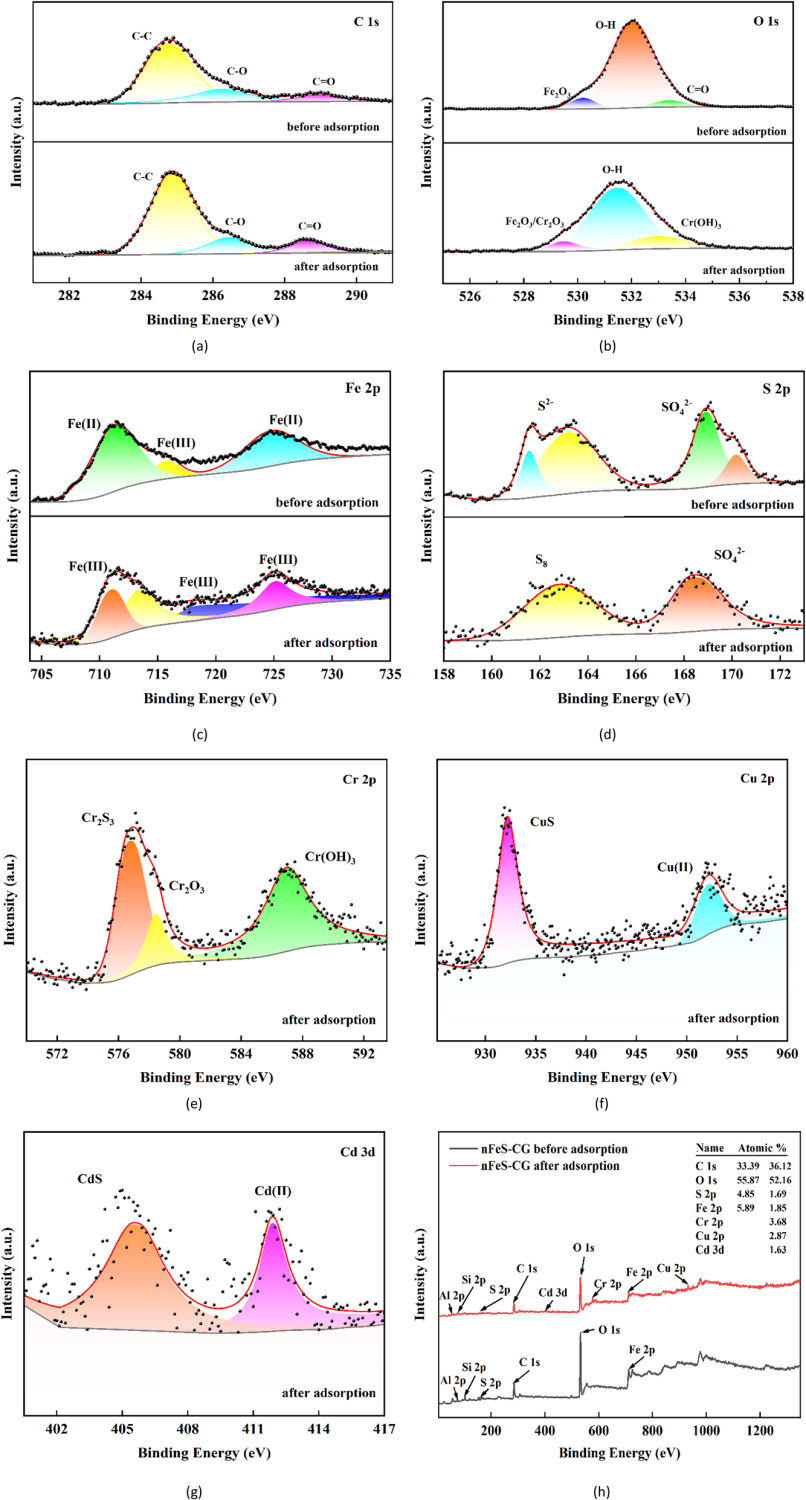
XPS spectra of nFeS-CG before and after adsorption of Cr(vi), Cu(ii) and Cd(ii).

In order to further analyze the removal mechanism of Cr(vi), Cu(ii) and Cd(ii) by nFeS-CG, the elemental composition and electronic structure before and after the reaction were characterized by XPS. It can be seen from the total spectrum of Fig. 18(h) that after nFeS-CG treatment of wastewater, the relative atomic content of Fe 2p decreased from 5.89% to 1.85%, and the relative atomic content of S 2p decreased from 4.85% to 1.69%, indicating that FeS participated in the chemical reaction. At the same time, Cr 2p, Cu 2p and Cd 2p appear in the nFeS-CG after adsorption, and the relative atomic content is 3.68%, 2.87% and 1.63%, respectively, indicating that nFeS-CG has good fixation ability for chromium, copper and cadmium.

As shown in Fig. 18(a), before the reaction, the binding energies of the C 1s peak of nFeS-CG at 284.7 eV, 285.7 eV and 288.4 eV are –C–C, –C–O and –C=O, respectively. After the reaction, the intensity of each peak increased to a certain extent, indicating that the redox reaction occurred on the reducing groups on the surface of coal gangue.

As shown in Fig. 18(b), the O 1s peak of nFeS-CG before reaction is at 530.2 eV (Fe_2_O_3_), 532.0 eV (–OH) and 533.4 eV (–C=O). After the reaction, the content of –OH decreased and the binding energy shifted, and the characteristic peak of Cr(OH)_3_ appeared at 533.1 eV, indicating that nFeS-CG reduced Cr(vi) to Cr(iii). The binding energy of Fe_2_O_3_ shifts to 529.5 eV, indicating that there are chromium hydroxides and oxides in the product, which is consistent with the XRD results.

As shown in Fig. 18(c), the Fe 2p peak of nFeS-CG before reaction is Fe(ii)–S at 711.5 eV and 724.9 eV, and the Fe_2_O_3_ characteristic peak at 715.9 eV, indicating that it is partially oxidized during storage. After the reaction, the characteristic peak of Fe(ii) disappeared and all of them were converted to Fe(iii), indicating that the redox reaction between Fe(ii) and Cr(vi) occurred.

As shown in Fig. 18(d), the S 2p peaks of nFeS-CG before reaction are S^2−^ characteristic peaks at 161.6 eV and 163.2 eV, and SO_4_^2−^ characteristic peaks at 168.9 eV and 170.2 eV, indicating that S^2−^ is partially oxidized, which is consistent with FTIR results. After the reaction, the characteristic peak of S^2−^ disappeared, and the characteristic peak of S^2−^ appeared at 162.9 eV. The characteristic peak of SO_4_^2−^ shifted to 168.5 eV, indicating that S_8_ and SO_4_^2−^ were the main products of S^2−^ oxidation.^46^

As shown in Fig. 18(e), the binding energies of Cr 2p characteristic peaks at 576.8 eV, 578.5 eV and 587.0 eV are Cr_2_S_3_, Cr_2_O_3_ and Cr(OH)_3_, respectively, indicating that most of Cr(vi) is reduced to Cr(iii) and fixed on the surface of nFeS-CG by precipitation.^47^

As shown in Fig. 18(f) and (g), after the reaction, the characteristic peak of Cu 2p is CuS at 932.2 eV, and the characteristic peak of Cu(ii) appears at 952.2 eV, indicating that Cu^2+^ combines with S^2−^ to form CuS precipitation, and ion exchange, chelation and precipitation reactions may occur. The characteristic peak of Cd 3d is CdS at 405.6 eV, and the characteristic peak of Cd(ii) appears at 411.9 eV, indicating that Cd^2+^ and S^2−^ are combined to form CdS precipitate, and ion exchange, chelation and precipitation reactions may occur.^48^ It indicated that Cu(ii) and Cd(ii) were successfully immobilized on nFeS-CG by sulfide precipitation.^49^

### 3.9 Adsorption mechanism analysis

**Fig. 19:**
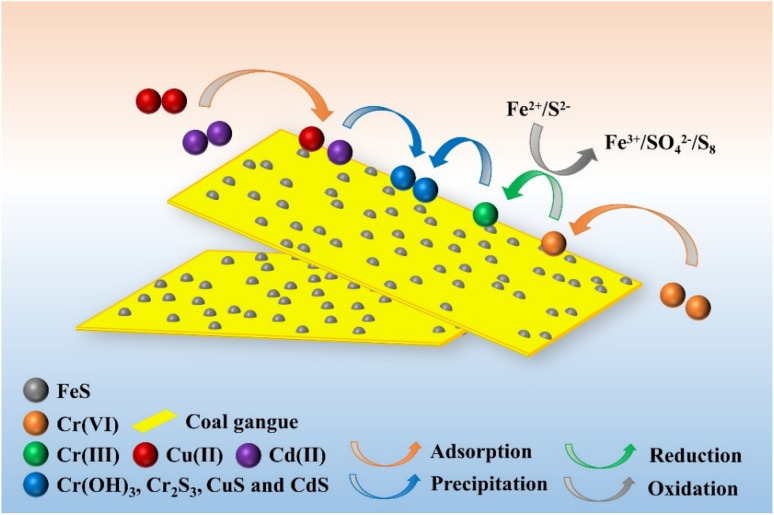
Adsorption mechanism diagram of Cr(vi), Cr(iii), Cu(ii) and Cd(ii) by nFeS-CG.

According to the analysis of material characterization and experimental results, the removal mechanism of Cr(vi), Cu(ii) and Cd(ii) by nFeS-CG is mainly adsorption, reduction and precipitation. The reaction mechanism is shown in Fig. 19.

Under acidic conditions, metal ions exist in the form of HCrO_4_^−^, Cu(ii) and Cd(ii), respectively. In the early stage of the reaction, under low pH conditions, high concentration of H^+^ makes nFeS-CG rapidly protonated, and the surface of the adsorbent is positively charged, which can quickly adsorb HCrO_4_^−^ ions in the solution through electrostatic attraction. Under acidic conditions, FeS on the surface of nFeS-CG can rapidly ionize Fe^2+^and S^2−^, and undergo redox reaction with Cr(vi) to form Cr(iii), Fe^3+^, S_8_ and SO_4_^2−^, thereby effectively reducing the toxicity of Cr(vi) and enhancing the overall removal efficiency.^50^ As a large amount of H^+^ is consumed, the pH of the system gradually increases, and nFeS-C can quickly adsorb positively charged Cu(ii) and Cd(ii) onto the surface of the adsorbent.^51^ At the same time, some S^2−^ ions will co-precipitate with Cr(iii), Cu(ii) and Cd(ii) to form insoluble precipitates such as Cr_2_S_3_, CuS and CdS on the surface of nFeS-CG. As the reaction continues, Cr(OH)_3_ precipitates appear on the surface of nFeS-CG.^52^

Combined with the experimental results of adsorption kinetics, it can be seen that the removal of Cr(vi), Cr(iii), Cu(ii) and Cd(ii) by nFeS-CG is mainly chemical adsorption. XPS characterization results show that Cr, Cu and Cd are finally fixed on nFeS-CG in the form of Cr(iii), Cu(ii) and Cd(ii) precipitates. Therefore, the removal mechanism of Cr(vi), Cu(ii), and Cd(ii) by nFeS-CG involves the synergistic effect of three pathways: (1) electrostatic adsorption and functional group interaction: surface functional groups of coal gangue provide initial binding sites through protonation-induced electrostatic attraction, coordination, and chelation; (2) redox reaction: nFeS reduces Cr(vi) to Cr(iii) with Fe^2+^ oxidized to Fe^3+^; (3) precipitation: Cr(iii), Cu(ii), and Cd(ii) form insoluble precipitates with S^2−^ or OH^−^. These processes collectively achieve efficient simultaneous removal. Coagulation was not involved due to the low concentration of Fe^3+^ and Al^3+^ in the solution, which failed to form effective coagulants under the experimental pH conditions. The relevant reaction is as follows:

14 FeS ↔ Fe^2+^ + S^2−^

15 FeS + H^+^ → Fe^2+^ + HS^−^

16 3Fe^2+^ + HCrO_4_^−^ + 7H^+^ → 3Fe^3+^ + Cr^3+^ + 4H_2_O

17 3S^2−^ + 2HCrO_4_^−^ + 14H^+^ → 3S + 2Cr^3+^ + 8H_2_O

18 3HS^−^ + 8HCrO_4_^−^ + 29H^+^ → 3SO_4_^2−^ + 8Cr^3+^ + 20H_2_O

19 3S^2−^ + 2Cr^3+^ → Cr_2_S_3(S)_

20 S^2−^ + Cu^2+^ → CuS_(S)_

21 S^2−^ + Cd^2+^ → CdS_(S)_

22 Cr^3+^ + 3H_2_O → Cr(OH)_3(S)_ + 3H^+^

## 4. Conclusion

This study presents a novel, cost-effective composite (nFeS-CG) that overcomes the agglomeration of nano-FeS and low adsorption capacity of raw coal gangue, achieving superior synchronous removal efficiency for Cr(vi), Cu(ii), and Cd(ii) in AMD. The mechanism, combining electrostatic adsorption, redox, and precipitation, provides a technical reference for the resource utilization of coal gangue and complex wastewater treatment. The optimum conditions for adsorption and removal of Cr(vi), Cu(ii) and Cd(ii) were determined: dosage 7 g L^−1^, reaction time 155 min. The adsorption process conforms to the pseudo-second-order kinetic model and the Langmuir model, indicating that it is monolayer adsorption and mainly chemical adsorption. The thermodynamic results show that the adsorption is an endothermic process, and the temperature rise is beneficial to the reaction. The leaching toxicity test shows that nFeS-CG has excellent immobilization ability for heavy metal ions and has good safety. The results of cyclic regeneration experiments showed that the adsorption capacities of nFeS-CG for Cr(vi), Cu(ii) and Cd(ii) were 10.69, 8.1 and 6.36 mg g^−1^, respectively, after three cycles of adsorption and desorption, indicating that nFeS-CG had good regeneration performance, good safety and economy. The removal mechanism of metal ions by nFeS-CG was elucidated by XRD, SEM, FTIR and XPS. The diffraction peaks of Cr(OH)_3_, Cr_2_S_3_, CuS and CdS appeared after the adsorption of nFeS-CG, and the deposition of particles appeared on the surface of the particles, indicating that nFeS-CG adsorbed heavy metal ions on the surface of the particles. Combined with XPS analysis, it can be seen that the particles are mainly Cr_2_S_3_, CuS and CdS precipitates formed by the reaction of nano-FeS with Cr(vi), Cr(iii), Cu(ii) and Cd(ii). The FTIR spectra after adsorption showed that the absorption peaks of –C=O and –C=C were enhanced, indicating that the reducing functional groups on the surface of coal gangue reacted with Cr(vi). In summary, the removal mechanisms of Cr(vi), Cu(ii) and Cd(ii) by nFeS-CG are mainly adsorption, redox and precipitation. The results showed that the removal rates of Cr(vi), Cu(ii) and Cd(ii) by nFeS-CG were increased, respectively, compared with FeS alone. It is confirmed that the coal gangue carrier can effectively improve the application performance of nano-FeS. However, there are some limitations in this study. The experimental use of simulated AMD may not fully reproduce the complexity of the actual mine wastewater. The recycling performance of nFeS-CG decreased after three cycles. The cost–benefit analysis of large-scale production of nFeS-CG has not been evaluated in detail. Future research should focus on on-site scale verification, material modification, mechanism quantification, cost optimization and long-term toxicity monitoring, so as to solve the existing limitations and promote practical application, and further optimize nFeS-CG into a sustainable and efficient adsorbent for heavy metal remediation of AMD.

## Author contributions

Xuying Guo: conceptualization, methodology, validation, formal analysis, writing—original draft preparation. Wei Sun: software, resources, data curation, visualization, writing—review and editing. Zilong Zhao: resources, project administration, supervision. Honglei Fu: project administration, supervision, validation. Xiaoyue Zhang: conceptualization, investigation, writing—original draft preparation. Fanbo Meng: investigation, project administration, supervision. Yanrong Dong: conceptualization, methodology, resources, supervision.

## Conflicts of interest

The authors declare that they have no known competing financial interests or personal relationships that could have appeared to influence the work reported in this paper.

## Data Availability

Data will be made available on request.
